# High cancer detection rate using cognitive fusion - targeted transperineal prostate biopsies

**DOI:** 10.1590/S1677-5538.IBJU.2016.0511

**Published:** 2017

**Authors:** Snir Dekalo, Haim Matzkin, Nicola J Mabjeesh

**Affiliations:** 1 Department of Urology, Tel Aviv Sourasky Medical Center, Sackler Faculty of Medicine, Tel Aviv University, Israel

**Keywords:** Magnetic Resonance Imaging, Prostatic Neoplasms, Biopsy

## Abstract

**Objective:**

MRI of the prostate improves diagnostic accuracy of prostate cancer. Different fusion approaches with transrectal ultrasound images are employed.

**Objective:**

To determine detection rate of prostate cancer in men undergoing transperineal MRI-based cognitive fusion biopsy.

**Materials and Methods:**

One hundred and sixty-four consecutive men underwent a multiple-core prostate transperineal biopsy. Univariable and multivariable logistic regression analyses were used to address the relationship between clinical parameters and prostate cancer detection rate.

**Results:**

One hundred and fourteen patients underwent mpMRI prior to the transperineal biopsy, 52 (45%) were diagnosed with prostate cancer, of them, 36 had Gleason score ≥7 (69%). Among these 114 patients, 82 had suspicious lesions on MRI, and 43 of them were diagnosed with cancer (52%). On multivariate analysis, the most significant independent predictive factors were PSA density (P<0.001) and suspicious MRI lesion (P=0.006). Men with a PSA density of more than 0.22 and a suspicious lesion on MRI had a detection rate of 78%. Detection rate among 50 patients with no MRI study prior to this biopsy was 26%.

**Conclusions:**

This study showed that among a group of mostly multi-biopsied patients, the presence of mpMRI lesions and high PSA density values helped to detect clinically significant prostate cancer using cognitive MRI/TRUS fusion biopsies.

## INTRODUCTION

Tools to enhance accurate detection of clinically significant prostate cancer are frequently developed. These tools are supposed to help avoiding the shortcomings of conventional biopsy such as false-negative results or under diagnosis of aggressive cancer as well as overdiagnosis of insignificant disease.

Until recently, saturation (at least 24 cores) biopsy was considered a method of choice to improve prostate cancer-detection rate after previous negative biopsy series (1-3). Bott et al. (4, 5) developed the brachytherapy template-guided transperineal technique, which increased the detection rate of significant tumors located especially in the anterior zone of the prostate gland. This procedure still has a role in defining disease previously missed or under-diagnosed (6). Transperineal saturation biopsies require increased resources in comparison with standard local anesthetic transrectal biopsies. In contrast to the high rate of infections caused by the transrectal approach (up to 5%), the transperineal approach is more prone to acute urinary retention. Most importantly, it seems most would agree that the transperineal approach enables the examiner to get samples from prostate areas that are difficult, if not impossible, to sample by the transrectal approach (7).

Multiparametric magnetic resonance imaging (mpMRI) of the prostate has become recently a promising tool being increasingly used to improve the accuracy of prostate cancer detection. Suspicious lesions on mpMRI can guide targeted biopsy and allow better detection of clinically significant tumors and avoiding unnecessary repeated random biopsies (8-10).

Different approaches to the use of MRI in performing biopsies are currently employed. The most promoted one is via the use of dedicated hardware and algorithm-based fusion software (11-13). One can also perform the fusion based on cognitive appraisal of the location of the suspicious lesion seen on the different MR images on TRUS without any additional equipment (14, 15).

We hypothesized that the use of cognitive MRI/TRUS fusion transperineal template-guided biopsy enables better detection of clinically significant prostate cancer. In this cohort, most patients underwent multiple negative biopsies before they were referred to this biopsy.

In the present study, we evaluated the detection rate of cancer in the prostate gland in these men as well as the predictive factors for prostate cancer detection.

## MATERIALS AND METHODS

The Institutional Review Board approved this study and waived informed consent requirements. Between the years 2011-2015, 164 consecutive men underwent transperineal template-guided biopsy from six regional locations, multiple core biopsies from each region, extending from the base, mid-gland and apex (16, 17). Biopsies were taken randomly from all regions; otherwise there were suspicious lesions on MRI, biopsies were directed first to those lesions. For 20 patients whose lesions on MRI were characterized using the first version of Prostate Imaging - Reporting and Data System (PIRADS, (18)) methodology, all PIRADS ≥3 were considered suspicious.

Biopsies were performed by two senior urologists (HM and NM) with previous extensive experience in transperineal prostate saturation biopsies as well as transperineal brachytherapy implant in the operating room under general anesthesia with the patient in the dorsal lithotomy position (17, 19). All men received perioperative antibiotics and an enema. The setup was the same as that used by us for brachytherapy. Before biopsy, the prostate gland was scanned from the level of the proximal seminal vesicles/base of the prostate gland to the apex and prostate volume was determined and the region of interest (suspicious lesions) was projected (cognitively) according to the MRI study.

This cohort of men had a history of pre-study biopsies with 113 men with at least 2 prior biopsies (2-8 biopsies, 69%), 45 men underwent 1 biopsy, (27%) and 6 men with no prior biopsy (4%).

One-hundred fourteen patients performed mpMRI prior to the current biopsy. All MRI scans were performed with either 1.5 Tesla with endorectal coil (n=80) or 3 Tesla without endorectal coil (n=34). Senior radiologists evaluated suspicious lesions using T2-weighted imaging, diffusion-weighted imaging (DWI), and dynamic contrast-enhanced MRI.

These studies were reviewed before and during the procedure to help direct the biopsies on a cognitive basis to the suspicious MRI lesions and extra random cores were taken from all over the prostate gland as described above.

All patients who underwent prior biopsies had negative results except for 9 patients who were referred to this biopsy as part of their active surveillance program. All 9 had a Gleason score 6 prostate cancers.

The biopsy procedure was performed after thorough study of the mpMRI when available, with 18G, 20cm long Pro MagTM biopsy needles (Angiotech, Medical Device Technologies, Inc., Gainesville, FL, USA), which were placed transperineally through template apertures to correspond with the regional biopsy locations as described by Bott et al. (4). At least five biopsy cores, depending on prostate size, were obtained for each of six regional biopsy locations extending from the apex toward the base. Patients who underwent mpMRI prior to the biopsy had several targeted biopsies taken on a cognitive basis and only then the random saturation biopsies were obtained. Patients were instructed to continue oral antibiotics for another 72 hours.

Descriptive statistics of the study sample were used to summarize participant characteristics. The Student’s t-test was used for comparison of two means. Fisher’s exact test was used for two proportions. Backward likelihood ratio multivariate logistic regression analysis was used to identify predictors of prostate cancer and to build the prediction model. Classification and regression tree and χ^2^ automatic interaction detection (CHAID) methods were used to divide the predictors into categories on the basis of the cancer detection status (20). All tests were two-tailed and statistical significance was defined as a P <0.05.

## RESULTS

All 164 consecutive men who had transperineal template-guided biopsy were included in the study. Table-1 summarizes the clinical features of the evaluated men.

**Table 1:** Clinical features of 164 men who underwent prostate biopsies.

Characteristics	Total sample	Men performed MRI	Men did not perform MRI	P value*
No.	164	114	50	
Age (yrs); mean (SD, median)	65.18; (6.5, 65)	65.5 (6.8, 65)	64.3 (5.6, 64)	0.27
PSA (ng/mL); mean (SD, median)	14.3; (14.2, 11.2)	15.9; (16.3, 12)	10.6; (5.8, 9.6)	0.02
No. of prior biopsies mean; (SD, median)	2.1; (1, 2.2)	2.19; (1.2, 2)	1.87; (1.03, 2)	0.12
No. of cores taken during biopsy; mean (SD, median)	37.6; (4.9, 36)	37.7; (4.6, 36)	37.4; (5.5, 37)	0.7
Suspicious DRE; (No.,%)	23; (14)	18; (16)	5; (10)	0.46
Prostate volume (mL); mean (SD, median)	63; (32.1, 60)	60.8; (35, 57)	67.8; (23.5, 67)	0.2
PSA density**; mean (SD, median)	0.26; (0.18, 0.26)	0.3; (0.22, 0.3)	0.16; (0.07, 0.15)	0.001

**PSA =** prostate-specific antigen; **DRE =** digital rectal examination; **SD =** standard deviation.

* comparison between men who performed and who did not perform an MRI study.

** ng/mL/prostate volume (mL)

Adenocarcinoma was diagnosed in 65 men (40%). Of them, 42 (65%) had Gleason score ≥7. One hundred and fourteen patients underwent mpMRI prior to this biopsy, 52 (45%) were diagnosed with prostate cancer; of them, 36 had Gleason score ≥7 (69%). Among these 114 patients, 82 had suspicious lesions on MRI, 43 of them were diagnosed with cancer (52%). Only 9 patients out of the 32 patients with normal MRI findings were diagnosed with cancer (28%, P=0.02).

**Figure 1:**
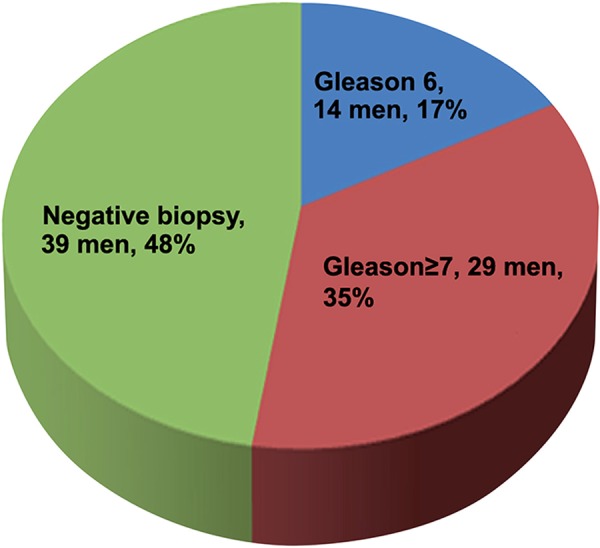
Cognitive fusion biopsy results of 82 men who had suspicious prostate Lesions on MRI.

**Figure 2:**
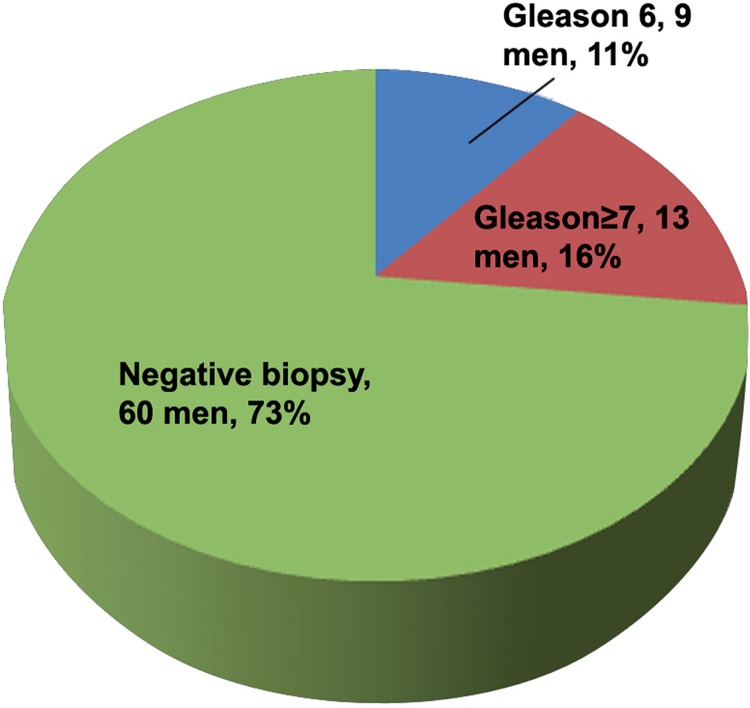
Transperineal biopsy results of 82 men without suspicious prostate Lesions on MRI.

Among 82 men with suspicious MRI lesion who underwent cognitive fusion biopsy 29 patients (35%) had clinically significant disease (Figure-1). On the other hand, only 13 patients (16%) had significant disease out of the 82 men with normal MRI and men who did not perform an MRI study (Figure-2, P=0.007).

Prostate cancer detection rate among patients with no mpMRI study prior to this biopsy was 26% (13 patients out of 50); of them 6 had Gleason score ≥7 (46%).

Cognitive MR fusion-targeted biopsies detected 82% of cancers in the region of interest whereas the rest of cancer were detected in the same side of the region of interest.

Logistic regression models were constructed to identify significant independent predictors of prostate cancer among 114 patients who underwent mpMRI prior to the biopsy. Factors that were evaluated as related to prostate cancer were age, PSA, PSA density, number of previous biopsies, number of cores taken during the biopsy, suspicious MRI lesion, prostate volume, suspicious digital examination. Because PSA density, PSA and prostate volume were highly correlated, the last two were not included in the regression model. The model identified significant association between PSA density, suspicious MRI lesion and prostate cancer (Table-2). The number of previous biopsies was nearly insignificant.

**Table 2:** Factors associated with prostate cancer detection among men who performed MRI.

P value	OR (95% CI)	Factor
< 0.001	1.104** (1.055-1.155)	PSA density*
0.047	0.625 (0.393-0.995)	No. of prior biopsies
0.006	4.986 (1.601-15.527)	Suspicious MRI lesion

*, ng/mL/prostate volume (mL)

**, per 0.01 increments of PSA density.

We next used the CHAID methodology to create a decision tree and found that patients with PSA density higher than 0.22 have 60.2% chance to be diagnosed with prostate cancer. When combining the existence of a suspicious MRI lesion, the detection rate increased to 78.4% (Table-3).

**Table 3:**
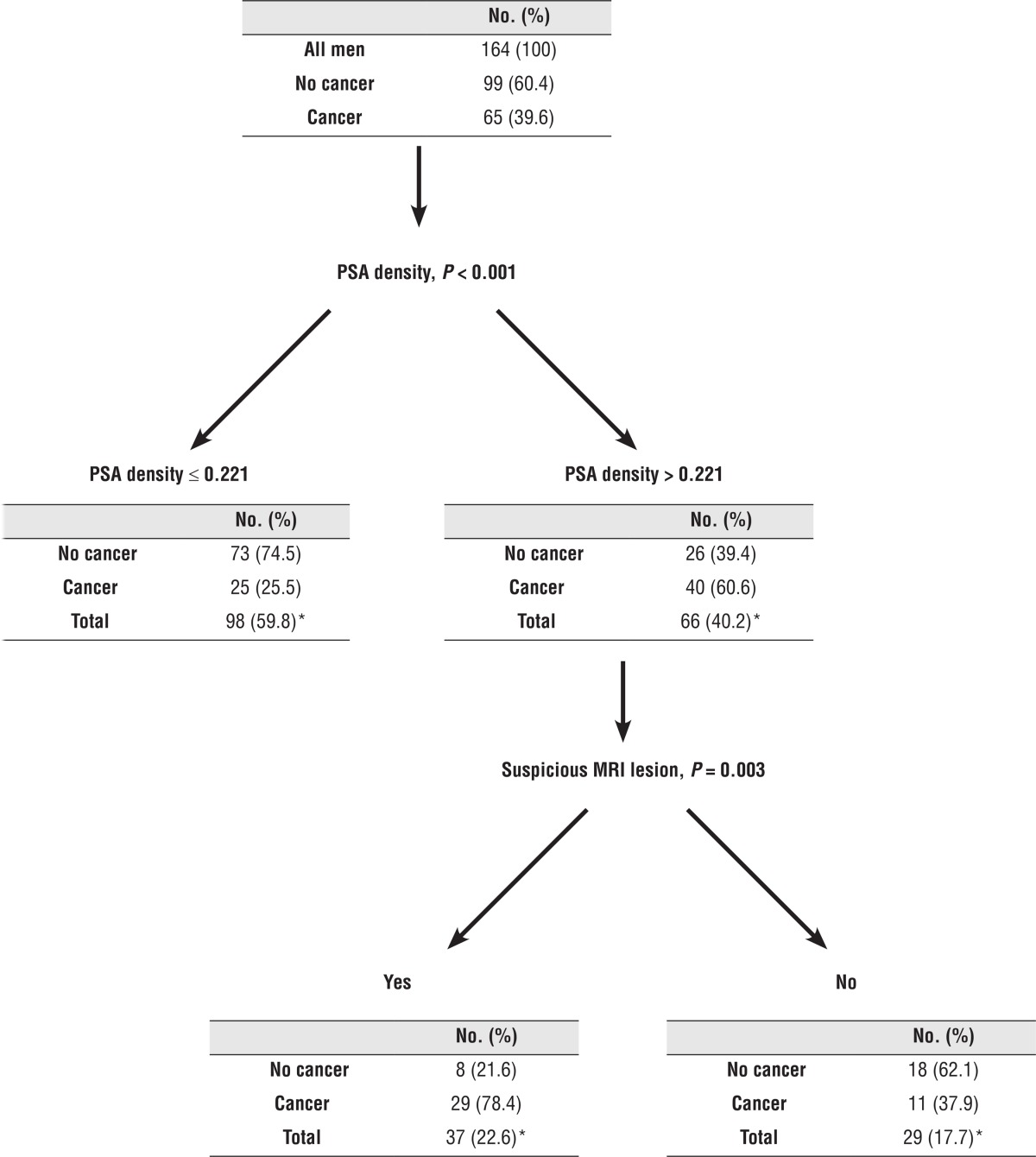
CHAIDS decision tree

*, percent relates to total number of patients (164).

## DISCUSSION

In this study, cognitive MRI/TRUS fusion targeted-biopsy enabled better detection rate of prostate adenocarcinoma and specifically clinically significant cancers in a highly pre-study biopsied population. Detection rate among patients with no MRI study prior to this biopsy was 26%, similar to already published data from other centers (16) as well as by ourselves (17). It is important to note that all patients were referred to us from other centers and the decision whether to perform MRI prior to the biopsy was not ours.

Men with a suspicious lesion on MRI had a detection rate of 52%. The presence of a suspicious lesion on mpMRI and PSA density were significant independent predictors for cancer detection on our multivariate regression analyses. Interestingly, men with a PSA density more than 0.22 and a suspicious lesion on MRI had a detection rate of 78% (Table-3).

Several studies evaluated the use of cognitive fusion transperineal biopsies. Kasivisvanathan et al. examined 182 men with a lesion suspicious for cancer on MRI and found that transperineal prostate biopsy cognitively targeted to these lesions detected clinically significant cancer in 57% (21).

Valerio et al. compared software vs. cognitive based targeted transperineal prostate biopsies and found that both methods were almost comparable with only slightly and not statistically significant better results using the software based approach (64% vs. 68% detection rate, respectively) (22).

Radtke et al. compared systematic transperineal saturation biopsies to magnetic resonance imaging targeted biopsy and showed that while detecting similar amounts of Gleason score 7 or greater tumors, the use of mpMRI/TRUS fusion mitigated the detection of lower grade disease (23).

The present study has several limitations. First, this is a retrospective study without any randomization and therefore, inherently contains biases regarding patient selection data. However, the fact that this is a consecutive series and none of the men were excluded strengthens our conclusions and their applicability in daily urological practice. Second, the fact that the two groups (with and without prior mpMRI) had significantly different PSA density values (although almost identical in other measures) did not allow us to use statistical tests when comparing these two groups. Third, unfortunately, PIRADS score was not constantly described in the MRI reports and the existence of suspicious lesions was analyzed by the radiologist in a binary manner. Last, comparison was not established against software based MRI-US fusion technique.

In conclusion, this study showed that among this group of mostly multi-biopsied patients, the presence of mpMRI lesions and high PSA density values helped to detect clinically significant prostate cancer using cognitive MRI/TRUS fusion biopsies.

In this era, software-based fusion technologies are getting more and more popular among urologists worldwide. Our study shows that urologists who are experienced with biopsies and brachytherapy using the transperineal approach can consider the cognitive fusion approach as a feasible and promising technique for increasing the detection of significant prostate cancer. Although cost effectiveness was not evaluated in this report, one can assume that obviating the need for high-tech equipment will also reduce costs.
